# Exploring reasons for variations in anxiety after testing positive for human papillomavirus with normal cytology: a comparative qualitative study

**DOI:** 10.1002/pon.5540

**Published:** 2020-09-21

**Authors:** Emily McBride, Laura A. V. Marlow, Kirsty F. Bennett, Selma Stearns, Jo Waller

**Affiliations:** ^1^ Department of Behavioural Science and Health Institute of Epidemiology and Health Care University College London (UCL) London UK; ^2^ Cancer Prevention Group School of Cancer and Pharmaceutical Sciences King's College London (KCL) London UK

**Keywords:** anxiety, cancer, cervical cancer, cervical screening, cytology, HPV, mental health, oncology, psychology, psycho‐oncology

## Abstract

**Objective:**

To explore reasons for variations in anxiety in women testing positive for human papillomavirus (HPV) with normal cytology at routine HPV primary cervical cancer screening.

**Methods:**

In‐depth interviews were conducted with 30 women who had tested HPV‐positive with normal cytology, including 15 with low‐to‐normal anxiety and 15 with high anxiety. Data were analysed using Framework Analysis to compare themes between low and high anxiety groups.

**Results:**

Several HPV‐related themes were shared across anxiety groups, but only highly anxious women expressed fear and worry, fatalistic cognitions about cancer, fertility‐related cognitions, adverse physiological responses and changes in health behaviour(s). In comparison to those with low anxiety, women with high anxiety more strongly voiced cognitions about the 12‐month wait for follow‐up screening, relationship infidelity, a lower internal locus of control and HPV‐related symptom attributions.

**Conclusions:**

Receiving an HPV‐positive with normal cytology result related to various emotional, cognitive, behavioural and physiological responses; some of which were specific to, or more pronounced in, women with high anxiety. If our observations are confirmed in hypothesis‐driven quantitative studies, the identification of distinct themes relevant to women experiencing high anxiety can inform targeted patient communications and HPV primary screening implementation policy.

## Highlights

- To our knowledge, this is the first qualitative study to explore anxiety in women testing human papillomavirus (HPV)‐positive with normal cytology at routine HPV primary screening.
- Our comparative qualitative design allowed thematic nuances to emerge between women who had experienced low versus high anxiety following their result.
- Only highly anxious women expressed fear and worry, fatalistic cognitions about cancer, fertility‐related cognitions, adverse physiological responses and changes in health behaviour(s).
- Highly anxious women also more strongly voiced cognitions about the 12‐month wait for follow‐up screen, reltionship infidelity, a lower internal locus of control and HPV‐related symptom attributions.
- These distinct themes can be used to minimise unnecessary anxiety through the development of evidence‐based patient communications at HPV primary screening.

## 1 BACKGROUND

Human papillomavirus (HPV) is a common sexually transmitted infection (STI), high‐risk types of which are responsible for virtually all cervical cancers. In 2019, the English National Health Service Cervical Screening Programme (NHSCSP) fully implemented routine HPV primary screening, where cervical cell samples are first tested for HPV and cytology (microscopic cell examination) is used to triage HPV‐positive results. Under HPV primary screening, women can test positive for HPV with normal cytology (HPV+/normal). Around 270,000 women in England (8.5% of those attending screening) are expected to receive this result each year.^1^ Due to the absence of cytological abnormalities, an HPV+/normal result carries a very low absolute risk of cervical cancer; however, given that HPV has been detected, relative risk is higher and women are recalled early for repeat screening at 12 months. Most HPV infections clear naturally within 18 months (65%),^2^ and women are only referred to colposcopy after they test HPV+/normal three consecutive times at 12‐month intervals.^1^

Despite the low cancer risk associated with testing HPV+/normal cytology, it is increasingly clear that, as a group, these women experience higher short‐term anxiety than those with normal results^3^, ^4^; as well as elevated psychosexual distress for up to 12 months.^4^, ^5^ For most, anxiety remains in the normal range, but nearly a quarter experience clinically significant anxiety for reasons that remain largely unclear.^3^ Psychological responses have been well documented in women testing HPV‐positive with abnormal cytology (where cancer risk is greater) but have been less well explored in women testing HPV+/normal at routine screening.^4^ Misinterpreting an HPV+/normal result could cause unnecessary anxiety if women overestimate their risk of cervical cancer. The positive (HPV) and negative (cytology) terminology may also evoke confusion. In the absence of abnormal cytology, some women may focus more on the sexually transmitted aspects of HPV which could lead to concerns about sex/relationships. Importantly, the 12‐month follow‐up interval means no routine clinical contact in the interim, which could cause and/or intensify psychological consequences.

The aim of this study was to explore reasons for variations in anxiety in women testing HPV+/normal at routine HPV primary cervical screening. In‐depth interviews were conducted with women purposively sampled from a larger quantitative study to compare those scoring low‐to‐normal versus high for anxiety, shortly after receiving an HPV+/normal screening result.

## 2 METHODS

Women aged 24–63 who had tested positive for HPV with normal cytology were recruited to take part in a qualitative study through two NHSCSP HPV primary screening sites in England. The in‐depth interviews were conducted with women who had taken part in a survey assessing their anxiety scores (see: doi.org/10.1186/ISRCTN15113095), who did not report a current anxiety disorder. Women were purposively sampled to compare the experiences of those with low‐to‐normal versus high anxiety (indicated by a score of ≤38 vs. ≥49 on the S‐STAI‐6,^6^ respectively). Where possible, they were also sampled to represent a range of demographics (e.g., age, ethnicity and education). Approvals were received from the Health Research Authority Research Ethics Committee (18/EM/0227), Confidentiality Advisory Group (18/CAG/0118) and Cervical Screening Research Advisory Committee (ODR1819_005).

If women completed the survey, they could opt‐in to be considered for an interview. The in‐depth semi‐structured interviews followed a topic guide (see Supporting Information 1) developed using the existing literature,^7^, ^8^ and grounded in relevant psychology theory (including Leventhal's Common‐Sense Model of Illness and Cognitive Behavioural Theory^9^, ^10^). Interviews took place face‐to‐face between 28th June and 31st August 2019, were audio‐recorded and transcribed verbatim.

Data were coded using the qualitative analysis software NVivo 12 and a 10% check indicated good inter‐rater reliability (Kappa = 0.91). The codes were summarised in a framework matrix to allow for theme comparisons between participants who had scored low‐to‐normal versus high for anxiety. Framework Analysis^11^ was chosen because it facilitates comparisons within and between cases.^12^ Greater methodological detail is available (see S2).

## 3 RESULTS

Interviews were conducted with 30 women, including 15 with low‐to‐normal anxiety (median score of 26.7, range: 20.0–36.7) and 15 with high anxiety (median score of 63.3, range:53.3–80.0). Table 1 displays a summary of participant characteristics.

**TABLE 1:** Participant characteristics and demographics overall and by anxiety group

	Overall sample (*N* = 30)	Low anxiety (*N* = 15)	High anxiety (*N* = 15)
Anxiety score (median, range)	45.0 (20.0–80.0)	26.7 (20.0–36.7)	63.3 (53.3–80.0)
Age in years (median, range)	37.5 (24.0–63.0)	48.0 (26.0–63.0)	33.0 (24.0–63.0)
Education (*N*)			
Below degree	15	7	8
Degree or higher	15	8	7
Ethnicity (*N*)			
White	22	11	11
Black	4	3	1
Asian	3	1	2
Mixed/multiple	1	0	1
Relationship status (*N*)			
Partner	23	12	11
No partner	7	3	4
Index of multiple deprivation^a^ *(N)*			
Most deprived (deciles 1–5)	19	9	10
Least deprived (deciles 6–10)	11	6	5
HPV with normal cytology result (*N*)			
1^st^ result	21	9	12
2^nd^ or 3^rd^ result^b^	9	6	3

Abbreviation: HPV, human papillomavirus.

^a^ Index of multiple deprivation is a multidimensional marker of area‐level deprivation, based on residential postcode.

^b^ Women had tested HPV+/normal for a 2^nd^ or 3^rd^ consecutive time at their 12‐month recall at HPV primary screening.

Interviews lasted on average 46 min (range: 24–75 min). Women completed their anxiety questionnaire on average 11.5 days (range: 6–53 days) after receiving their test result and attended the interview on average 35.5 days after their result (range: 22–76 days).

### 3.1 Summary of themes

Women's reactions to receiving test results covered five themes: (1) emotional response, (2) cognitions related to HPV, (3) behaviours, (4) disclosure of result and (5) physiological response. Differences between low and high anxiety groups are highlighted throughout. Quotes are reported with participant number (P) and by anxiety group (low as LA; high as HA).

**FIGURE 1:**
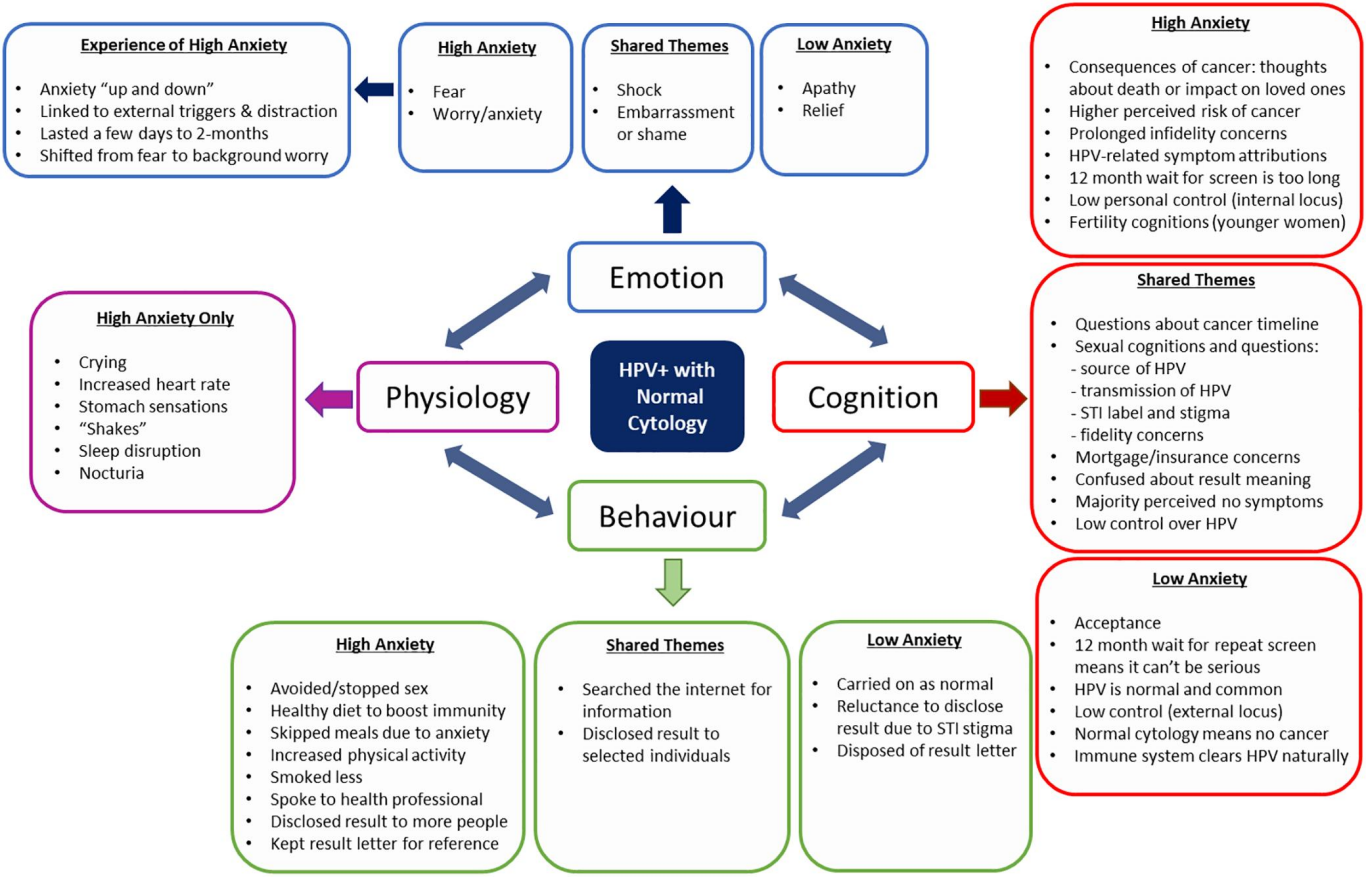
Thematic comparisons between women with low‐to‐normal versus high anxiety

See Figure 1 for an overview of the thematic comparisons between low versus high anxiety.

#### 3.1.1 Emotional response

Adverse emotional responses were described mainly by women with high anxiety. Many described experiencing fear or worry shortly after receiving their results, which often related to the development of cervical cancer and/or its potential impact on their family.

It's that panic. You think, oh my goodness. You don't want it [cancer] to happen to you and your family. You want ‐ you want that bubble, you want… to be able to protect ‐ protect it and protect them. (P25, HA)

The period between receiving a result and attending the interview was described as ‘up and down’ (P7, HA), sometimes linked to external triggers (e.g., cancer on TV) or lack of distraction. The period of highest anxiety was reported as lasting between a few days and a couple of months. However, for some, the result remained an ‘underlying anxiety’ (P2, HA).

The first couple of months it was there. Um then it sort of faded… (P20, HA)

Both anxiety groups described ‘shock’ or ‘surprise’ immediately after their result because they had no symptoms, had been vaccinated, or were unaware they were being tested for HPV. After the initial shock, some women in the low anxiety group reported little concern or ‘relief and reassurance’ (P6, LA).

Some women receiving an HPV+/normal result for the second or third time were ‘more relaxed’ because they knew what to expect, whilst others felt worse because their body was not successfully fighting HPV. After a third result, two women felt ‘reassured’ to be offered further investigation (colposcopy).

#### 3.1.2 Cognitions about HPV

Cognitions about HPV covered six subthemes: (1) understanding of result, (2) cervical cancer and the aetiology of HPV, (3) 12‐month screening interval, (4) sexual impact, (5) symptom attributions and (6) other cognitions.

##### Understanding of result

Few women were aware they had been tested for HPV prior to receiving their result. Many were ‘confused’, ‘perplexed’ or ‘uncertain’ about its meaning. There was particular confusion around the combined positive (for HPV) and negative (for cytology) aspect, as well as what ‘cytology’ or having an ‘infection’ meant.

So you're thinking well I've not got cancerous cells, I'm okay for three years, so you think yeah, thumbs up. But then, but you've got HPV so you might get cancerous cells. Do you understand what I mean? (P14, HA)

##### Cervical cancer and the aetiology of HPV

Most women were aware that sexual activity caused HPV. Others guessed that HPV was caused by poor hygiene or was a symptom of another condition. Most believed that HPV lasted 1–2 years; though a minority believed HPV would stay ‘forever’ or had been there ‘since birth’. Some women wanted to know whether they had ‘high‐risk’ HPV in order to assess their cancer risk. Highly anxious women viewed themselves as medium‐to‐high risk of cervical cancer.

In the letter they obviously tell you that ‐ that 50 per cent of the women carry the virus, but only 70 per cent of those women develop cervical cancer… so you're thinking to yourself… basically I've got 70 per cent chance of developing cervical cancer. (P1, HA)

In contrast, most women in the low anxiety group reported feeling at ‘low’ risk of cancer and that their result was ‘not serious’. Low perceived risk was related to not having abnormal cells; HPV not being the direct cause of cancer; and HPV not causing problems when dormant or detected early.

Consequences related to developing cancer and its future impact were shared subthemes for both groups; however, these cognitions were prominent in highly anxious women. Some described thoughts about death and cancer treatment.

Because you start thinking, ‘Is my life sort of coming to…’ because I don't know, if I do get it, will my body be able to err, go through the therapy and will I be, will I survive or not survive? (P26, HA)

Several highly anxious women discussed the potential impact of cancer on their children or partner. In contrast, women in the low anxiety group explained that there was no point focussing on cancer because ‘worrying about it isn't going to help’ (P18, LA).

When the kids do something that makes me proud or, or something like that. And then I'll think oh, what if I wasn't here to see it. (P5, HA)

Several women wanted to find a cure for HPV and some had thought about treatments they could undergo to avoid cancer (e.g., hysterectomy).

Normally you have a virus, you take some medication and you kill the bloody thing. Why am I not being given any medication? (P1, HA)

Regardless of anxiety group, nearly all women reported ‘no control’ or a lack of control over HPV. Women with low anxiety expressed that they did not need to exert control because HPV clearance and transmission depended on external factors, so they were ‘accepting’ or ‘calm’ about it. Some of these women suggested that viral infections were inevitable or they took the fatalistic view, ‘if your time's up, your time's up’ (P28, LA).

At the moment I don't really feel like I've got any particular control. Um I don't really feel like I need to have control of it. It just exists. (P21, LA)

In contrast, highly anxious women reported unease about their lack of control and described feeling ‘lost’. One woman described HPV as a ‘ticking time bomb’ because she could not control its progression to cancer (P14, HA).

I also feel like it gives you that feeling of helplessness that you can't do anything to make it better. I don't really like that feeling. (P24, HA)

Most highly anxious women sought ways to exert control. Some searched for treatments or changed their behaviours (described under ‘behaviours’). One woman highlighted that there was merit in ‘trying to do something, regardless of whether it actually does anything’ (P5, HA). A minority of women across both groups believed that they had ‘a lot’ of control over HPV via attending their follow‐up screen, monitoring themselves for cancer symptoms, and controlling the transmission of HPV to others. A couple were ‘confident’ their body would fight HPV.

##### 12‐Month screening interval

In the UK, women who receive an HPV+/normal result for the first or second time at HPV primary screening are recalled for a 12‐month follow‐up screen. Whereas many women in the low anxiety group believed the 12‐month wait was ‘normal’, nearly all highly anxious women wanted to re‐attend earlier.

I think it's a long time to wait, twelve months. Yeah, maybe six months or three months, but I think twelve months is a long time to wait. (P4, HA)

Some wanted more information on the rationale for the interval and questioned the decision‐making of doctors/policymakers, with suggestions it could be a ‘financial’ decision. Many highly anxious women questioned whether cancer might develop before their next screen. In contrast, a few women in the low anxiety group believed the 12‐month interval implied that HPV was not serious.

I feel like I've got the you know, sort of sword of Damocles on top of my head. I'm waiting for 12 months to know if something wrong is happening or not so it's all a bit…I find the 12 month wait, that's gonna be horrendous. (P1, HA)

I thought, ‘If it's anything more serious than that, they won't, they'd invite me in sooner’. (P9, LA)

A few women had already requested an earlier screen from their GP or were considering getting screened privately. One explained that she was more ‘accepting’ of the wait after she was informed her body can take up to 2‐years to clear HPV.

##### Sexual impact

Cognitions about the sexual impact of HPV mainly centred round the source of infection and timeline, transmission, the STI label and relationships/infidelity.

Several women reported feeling ‘in a fog’ (P6, LA) about who had given them HPV and when. Some were confident HPV was from their current partner whilst others were unsure.

Many questioned whether HPV was definitely an STI, whilst some believed HPV was ‘not an actual STI’ (P30, LA). Others wanted to know whether HPV could be passed on; condoms are needed; re‐infection can occur between partners; and whether the act of sex as opposed to sexual contact caused HPV. The STI label was reported as ‘dirty’ by some women, as well as ‘oppressive’ (P4, HA) and ‘nasty’ (P28, LA). Some associated HPV with sexual promiscuity.

Thoughts about potential infidelity were common and appeared more pronounced in highly anxious women. Many women reported believing that their (ex)partner may have been unfaithful; though, most no longer believed this at the time of interview.

I think I know that he hasn't been unfaithful. I think I do know that. I now appreciate this can happen and be in my body. I could have done this to myself 20 years ago. (P20, HA)

Infidelity continued to be an issue for some highly anxious women by adding an element of mistrust or disruption to their relationship. A couple thought that their partner may not want to engage in sex if they knew HPV was an STI.

If he thinks that I could give him something then he's… he can rightly not want to do it [sex], which is completely fine but then obviously that would affect our relationship. (P24, HA)

##### Symptom attributions

HPV was widely seen as asymptomatic but some highly anxious women attributed symptoms to the virus, including: the development of a fibroid in the womb; a urinary tract infection; breast milk production; previous genital warts; and thrush. One woman with low anxiety attributed flu‐like symptoms and weight gain to HPV. Some women across both groups mentioned symptoms but were unsure if they were connected to HPV, including irregular bleeding, cramp pain, cold sores, fallopian tube pain, cystitis, bleeding after sex, and bladder leaks.

##### Other cognitions

Fertility‐related consequences were mentioned by younger women in the high anxiety group. The HPV vaccine was also discussed and was linked with annoyance about not being offered it by those with high anxiety. Two women discussed the consequences of HPV on their health and mortgage insurance. One had been advised by her insurer that she needed to formally declare her second HPV+/normal result on her mortgage.

And so he went back to the insurance company and said should she put this down… and their answer was if it was the first one, no ‐ but now she's had two, yes. And we will not cover her for any treatment. (P28, LA)

#### 3.1.3 Behaviours

Only women with high anxiety reported changing their behaviour due to HPV. Some reported avoiding sexual intercourse or using condoms. A few attempted to boost their immune system with vitamin supplements, changes to diet, and exercise. One woman reported reducing smoking and another described vaping more to deal with the stress of HPV.

We've not had any sexual intercourse since I got the letter. (P18, HA)

For like the first month I was on this really healthy exercise and eating hype to boost my immune system! That was purely the h‐ HPV because I thought, oh, my immune system needs to fight it. (P24, HA)

Women were also asked what they did immediately after they received their result. Many reported using the Internet to search for information on HPV; and highly anxious women described this most extensively, stating it was often ‘unhelpful’. Women with low anxiety usually reported putting their result letter to one side, ‘skim reading’ it (P2, LA), or getting on with their day. Some women described using distraction (e.g., activities or work) to avoid thinking about their result.

#### 3.1.4 Disclosure of result

Seeking social support was described as a coping strategy to help deal with HPV. Nearly all highly anxious women reported disclosing their result to at least one person; though some delayed disclosure or did not tell certain individuals. Non‐disclosure in this context was often because women did not want to burden loved ones.

I just don't really want to worry her [sister] about this kind of thing. (P1, HA)

In the low anxiety group, the decision to disclose was mixed. A few women stated that they did not tell anyone because they were not concerned. Those who did disclose sometimes omitted certain information (e.g., the sexually transmitted aspect) due to embarrassment, not wanting to be viewed as ‘promiscuous’ (P22, LA), or viewing their result as ‘personal’ (P8, LA). Two women were contemplating whether to disclose their result to a partner.

I think I just tried to put it out of my head and I was a bit embarrassed so I never even discussed it with anyone. (P15, LA)

#### 3.1.5 Physiological response

Physiological responses were exclusive to highly anxious women. Soon after their result, some reported crying, sensations in their stomach, and/or sleepless nights. Others described bodily sensations such as shaking and increased heart rate, and nocturia. One reported that she lost her appetite due to her anxiety.

## 4 DISCUSSION

Our findings advance the qualitative literature by exploring psychological response to testing HPV positive with normal cytology at routine HPV primary screening and identifying themes which may be specific to women with high anxiety. Only highly anxious women expressed fear and worry, fatalistic cognitions about cancer, fertility‐related cognitions, adverse physiological responses, and changes in behaviour(s). In comparison to those with low anxiety, they more strongly voiced cognitions about the 12‐month wait for follow‐up screening, reltionship infidelity, a low internal locus of control and HPV‐related symptom attributions.

Similar to other studies, we found testing positive for HPV was linked to cognitions about cervical cancer and feelings of fear and worry.^3^ In our study, cancer‐related cognitions appeared to be the most dominant theme and primary concern for highly anxious women. In particular, these women often focussed on the consequences of cancer and expressed cognitions about undergoing cancer treatments or leaving loved ones behind. Further, many highly anxious women considered themselves to be at medium‐to‐high risk of cervical cancer. In particular, the normal cytology aspect of an HPV+/normal result indicates very low short‐term cancer risk and should therefore, in theory, offer reassurance. However, nearly all women focussed on the HPV‐positive aspect of their result and gave little or no attention to normal cytology. Instead, some incorrectly believed that HPV was the direct precursor to advanced cervical cancer. These findings help to interpret recent cross‐sectional research which found heightened anxiety associated with an HPV+/normal result at HPV primary‐screening.^3^ Targeted information in HPV+/normal result letters emphasising the very low short‐term cancer risk and explaining the relevance of normal cytology could improve women's understanding and help prevent unnecessary anxiety.

Linked to cancer‐related cognitions, many highly anxious women voiced concerns about the 12‐month wait for routine follow‐up, questioning whether cancer may develop in the interim. Given that 12‐month recall is specific to HPV primary screening, it is also important for screening policymakers to communicate the rationale for this interval in HPV+/normal result letters to help reassure women.

The sexually transmitted nature of HPV has previously been linked to feelings of stigma, shame, and embarrassment.^4^, ^7^, ^13^ To date, most studies have assumed that sexual concerns play a central role in the development of anxiety following an HPV‐positive result. Interestingly, however, we found that most sexual cognitions and related feelings of embarrassment were common to both anxiety groups. Relationship infidelity was the only subtheme which was more pronounced in women with high anxiety. Although they require confirmation using quantitative studies, our findings help tease out nuances pertaining to cognitive versus emotional responses to HPV. Longitudinal studies also support this notion given that psychosexual distress remains elevated for up to 12‐month, whereas general anxiety normalises within 3 months, indicating two distinct psychological pathways.^4^, ^5^

Typically, low perceived control is associated with poor health outcomes including adverse emotional response.^14^ In line with recent systematic review evidence for HPV,^4^ nearly all women in our study reported feeling that they had little or no control due to a lack of treatment or practical prevention methods for HPV. A novel finding was that highly anxious women appeared to focus on internal factors they could use to gain control (e.g., consuming multivitamins), in contrast to women with low anxiety who linked external factors (e.g., fate) to acceptance of HPV. These findings point to individual differences in the interaction between locus of control and coping styles which, in the absence of a viable solution for HPV, may drive feelings of anxiety.

HPV is asymptomatic, yet some highly anxious women believed or questioned whether certain idiosyncratic symptoms may be HPV‐related. Healthcare professionals and screening information materials should highlight the asymptomatic nature of HPV, while encouraging women to monitor for specific cervical cancer symptoms (e.g., unusual bleeding, pain from sex).

Fertility‐related cognitions associated with an HPV‐positive result have also been identified in previous studies.^7^, ^13^ In our study, although a relatively minor theme, this was specific to younger women with high anxiety. General practitioners could provide reassurance about fertility to younger women who have received an HPV+/normal result.

Few studies have explored physiological and behavioural responses to HPV, with the exception of some general sexual behaviours.^15^, ^16^ We incorporated these constructs into our topic guide, and several anxious women reported experiencing physiological sensations shortly after receiving their result (e.g., crying, shaking, stomach sensations), as well as changing their behaviour(s) due to HPV (e.g., stopping sex, vitamin consumption and avoiding cigarettes). Behavioural and physiological factors should be incorporated into future cervical screening evaluations to assess their relevance and the full psychological impact of receiving HPV‐positive results.

### 4.1 Study limitations

Recruitment was linked to routine clinical management at HPV primary screening, ensuring a diverse and well‐characterised sample. However, due to the relatively small numbers within each demographic group, we were unable to explore intersections between demographics and anxiety. We were able to calculate the time (days) between women receiving their result and attending interview, which ranged from 22–76 days. It is possible that this variability in time from result may have introduced heterogeneity in women's recall of events and/or experiences of anxiety. Finally, although we excluded women who reported a current anxiety disorder, we did not measure anxiety scores prior to HPV primary screening, meaning we could not determine whether receiving an HPV+/normal result was the primary source of their anxiety.

### 4.2 Clinical implications

To date, cervical screening patient communications and public health campaigns aimed at minimising adverse psychological impacts have wholly based their content on population research. Our findings begin to build an evidence‐base for the development of specific messages targeting the concerns of highly anxious women, which could be included in standard HPV‐positive results letters or covered in training for sample‐takers or GPs who discuss HPV results with women.

## 5 CONCLUSION

Receiving an HPV‐positive with normal cytology result related to various emotional, cognitive, behavioural, and physiological responses; some of which were specific to, or more pronounced in, women with high anxiety. To avoid unintended consequences for women attending HPV primary screening (e.g., unnecessary anxiety and/or adverse behavioural impacts), these distinct themes should be tested in hypothesis‐driven quantitative studies and used to guide the development of evidence‐based patient communications and screening implementation policy.

## CONFLICT OF INTEREST

The authors declare no conflicts of interest.

## AUTHOR CONTRIBUTIONS

Emily McBride conceived the study. Emily McBride and Selma Stearns collected the data. Emily McBride, Jo Waller, Laura A. V. Marlow and Kirsty F. Bennett analysed and interpreted the data. Emily McBride drafted the manuscript. All authors contributed to the final version of the manuscript.

6

### DATA AVAILABILITY STATEMENT

The data that support the findings of this study are available on request from the corresponding author. The data are not publicly available due to privacy or ethical restrictions.

## Supporting information

Supplementary Material

Supporting Material 2
