# Supplementary material for: Exploring reasons for variations in anxiety after testing positive for human papillomavirus with normal cytology: a comparative qualitative study
Source: Psychooncology. 2020 Sep 21;30(1):84–92. doi: 10.1002/pon.5540 (PMC8436740; doi:10.1002/pon.5540)
Supplement: Supplementary file 1 — Supplementary Material [file PON-30-84-s002.docx]

**Supplementary File 1 - Topic Guide**

1. **Introduction – Have you taken part in a research project or interview before?**

Information, Consent, Demographics, Introductions, Structure, Voucher, Confidentiality, Questions

1. **Can you tell me a bit about yourself?**
2. **You recently attended for cervical screening…**
   1. Can you remember how long ago that was?
   2. Have you been for cervical screening much in the past before that?
   3. Thinking about your most recent result … When did you get it? Was there much of a wait between getting screened and the result arriving?
   4. Can you remember what your test result said?
3. **How well do you feel you understand what your result means? HPV? With Normal cells?**
4. **I want you to think about when you opened your letter and saw your result. How did you react?**
   1. How did you feel? What emotions?
   2. What thoughts went through your head?
   3. Did you notice any changes in your body?
   4. What did you do immediately afterwards? Anything else?
5. **Can you compare the way you felt when you saw your result to anything similar that you’ve experienced?**
6. **What about now? Have your feelings changed much? More or less strong?**
   1. If so, when did it change?
   2. Why do you think that is?
   3. If relevant, what are you most worried about?
7. **If you had to compare the way you feel now about having HPV to a similar situation, what would it be?**
8. **Have you told anyone about your result?**
   1. Do you usually talk to people about your health? The same people? Health professionals?
   2. Do you find it helpful?
9. **Has your result impacted on any other area(s) of your life that we haven’t spoken about?**
10. **More generally, in your everyday life, how often would you say you feel stressed or anxious?**
11. **Beliefs about HPV:**
    1. What do you think caused your HPV?
    2. How much control do you feel you have over your HPV?
    3. How long do you think you will have HPV for?
    4. How much do you think cervical screening can help?
12. **Do you experience any symptoms that you think might be related to your HPV?**
13. **How likely are you to go back to cervical screening again in 12 months?**
14. **What did you think of the NHS letter and information that you got?**
    1. Wording – anything that stuck out?
    2. Anything particularly good?
    3. Anything particularly bad?
    4. Anything that could have helped?
15. **Anything that I’ve missed that you think is important or you want to add about anything that we’ve discussed?**
