# Supplementary material for: Exploring reasons for variations in anxiety after testing positive for human papillomavirus with normal cytology: a comparative qualitative study
Source: Psychooncology. 2020 Sep 21;30(1):84–92. doi: 10.1002/pon.5540 (PMC8436740; doi:10.1002/pon.5540)
Supplement: Supplementary file 2 — Supporting Material 2 [file PON-30-84-s001.docx]

**Supplementary File 2 – Extended Method**

Potential participants were identified by NHS staff at two large cervical screening sites in England (North West London and Greater Manchester NHS Foundation Trusts). Eligible women were mailed a study information pack to their home address shortly after receiving their result letter, which contained a questionnaire assessing state-anxiety (using the S-STAI-6) as part of a larger cross-sectional survey (further details: doi.org/10.1186/ISRCTN15113095). If women completed the survey, they could opt to be considered for an interview. Interview participants were purposively sampled to represent 15 women with low-to-normal anxiety and 15 women with high anxiety. Where possible, women were also sampled to represent a range of ages, ethnicities, educational attainment, and marital statuses. If selected to take part, women were provided with further information (verbal information, information sheet, written consent form) and were offered a £40 gift voucher, plus reimbursement for travel expenses.

The in‐depth semi-structured interviews followed a topic guide (see S1) developed using the existing literature and grounded in relevant psychology theory, including Leventhal’s Common-Sense Model of Illness and Cognitive Behavioural Theory. For example, the topic guide reflected an assessment of emotions, cognitions, behaviours and physiological responses, in line with Cognitive Behavioural Theory (see S1; questions 5a-d). It also covered Leventhal’s illness perceptions by including questions on perceived cause, controllability (personal and treatment control), consequences, and symptoms of HPV (see S1; questions 12a-d and 13). The areas covered in the topic guide were piloted with members of the project steering group and feedback was integrated from two Patient and Public Involvement (PPI) representatives. The interviews began with questions about the participant's experience of cervical screening and receiving their test result. The rest of the interview was driven by responses to this question, and also included questions about emotional and physiological response, cognitions, behaviours, perceived understanding, and disclosure of result. Interviews were carried out by a researcher trained in qualitative research methods and psychological assessment (EM). Interviews took place face‐to‐face between 28^th^ June 2019 and 31^st^ August 2019 and were audio-recorded and transcribed verbatim with participant identifiers removed. Emerging themes from the transcripts were noted whilst conducting the interviews, and some additional questions were iteratively incorporated as the interviews progressed. All women provided written informed consent before the start of the interview and were debriefed at the end.

In addition to demographic information provided by participants in the survey (age, ethnicity, educational attainment, marital status, mental health diagnoses), some data was obtained from their clinical records (test result, deprivation score, NHS site, date of screen, and anticipated date of test result delivery).

Data were coded using the qualitative data analysis software NVivo 12. EM and KB read all the transcripts, and JW and LM read 3-5 transcripts each. EM developed the initial codes and preliminary thematic framework and then KB, LM and JW independently reviewed them. All codes were discussed until there was consensus on the final thematic framework, which was refined iteratively. The interview transcripts were uploaded to NVivo and EM coded all transcripts. KB independently coded 10% of the transcripts (n= 3) to check the inter-rater reliability of the framework which was good (Kappa = 0.91).

Once all the data had been coded in NVivo, it was then summarised in a framework matrix to allow for comparison of themes between participants who had scored low-to-normal vs. high for anxiety. The framework matrix used rows for participants and columns for themes. Framework Analysis was chosen because it facilitates comparisons within and between cases.
